# Supplementary material for: The lncRNA Caren antagonizes heart failure by inactivating DNA damage response and activating mitochondrial biogenesis
Source: Nat Commun. 2021 May 5;12:2529. doi: 10.1038/s41467-021-22735-7 (PMC8099897; doi:10.1038/s41467-021-22735-7)
Supplement: Supplementary file 1 — Supplementary Information [file 41467_2021_22735_MOESM1_ESM.pdf]

Supplementary Information

The lncRNA *Caren* antagonizes heart failure by inactivating DNA damage response and activating mitochondrial biogenesis.

Sato M, Kadomatsu T, Miyata K *et al*.

a

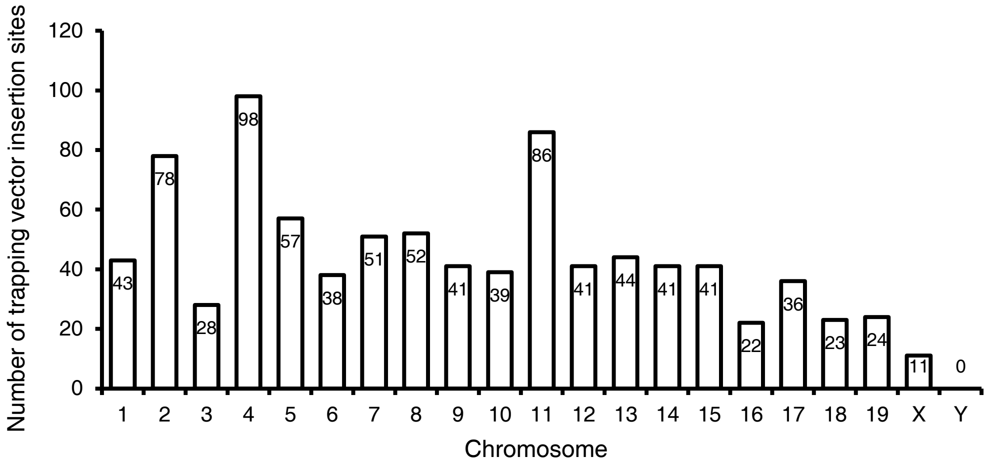

b

Clone ID	Chromosome	Strand	Trapped inserted position (NCBI37/mm9)	K4-K36 domain	Germline transmission
B186	17	-	29206273	+	+
T34	2	+	168834912	+	+
W148	13	+	98262197	+	+
W203	3	-	88326126	+	+
T167	8	+	59973653	+	+
W241	13	-	81782913	+	+
106	5	-	33831401	+	+
W147	15	-	5985073	+	+
T188	17	-	66800902	+	+
W115	11	+	51040338	+	+
W321	6	+	86479637	+	+
KBW264	14	+	76920458	+	+
T75	X	-	72995992	+	+

c

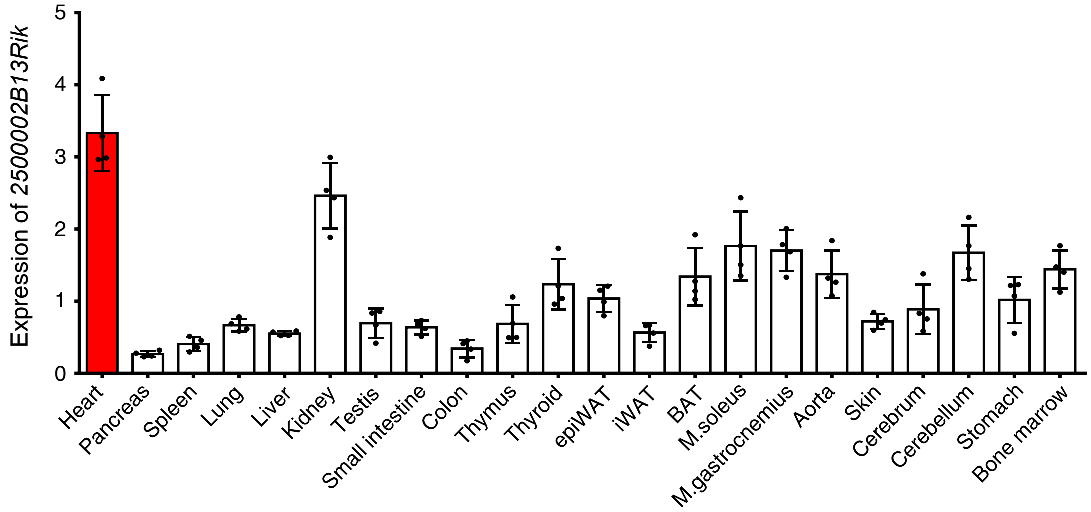

d

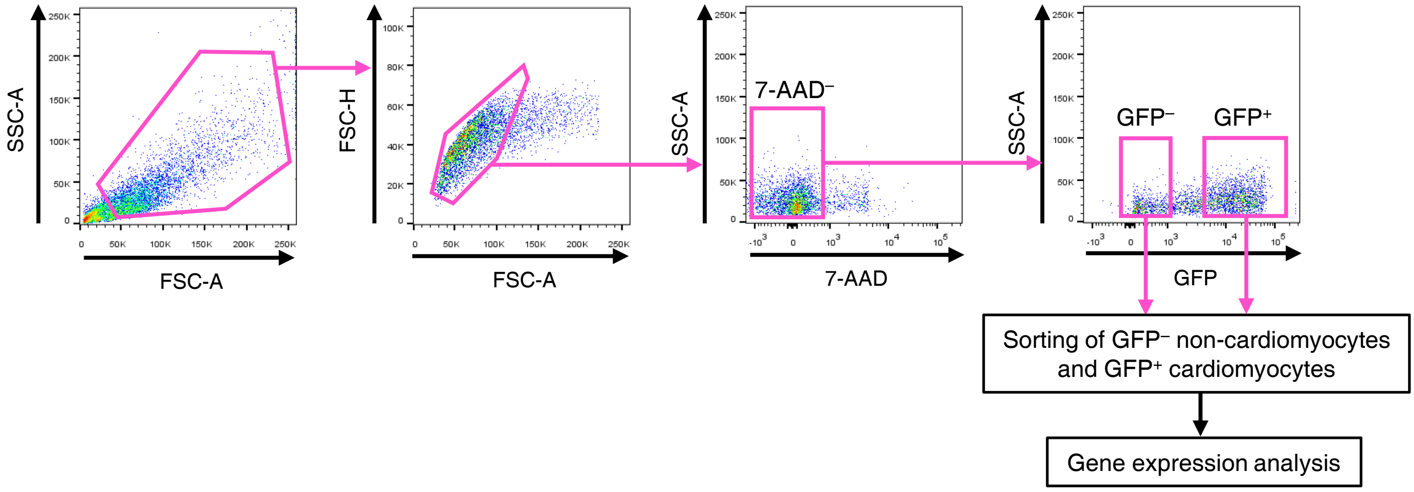

Supplementary Fig. 1 *2500002B13Rik* expresses in various tissues of adult mice.

a, Number of trapping vector insertion sites in murine chromosomes. **b**, List of 13 ES clones selected based on H3K4me3 and H3K36me3 histone modification (K4-K36 domain) profiles. **c**, Quantitative RT-PCR analysis of *Caren* in various tissues of 12-week-old WT mice (n = 4 each). Data are mean ± SD and all points. **d**, Gating strategy used to sort GFP^- non-cardiomyocytes and GFP^+ cardiomyocytes from heart tissues of *αMHC-EGFP* Tg mice for gene expression analysis (Fig. 1f). Source data are provided as a Source Data file.

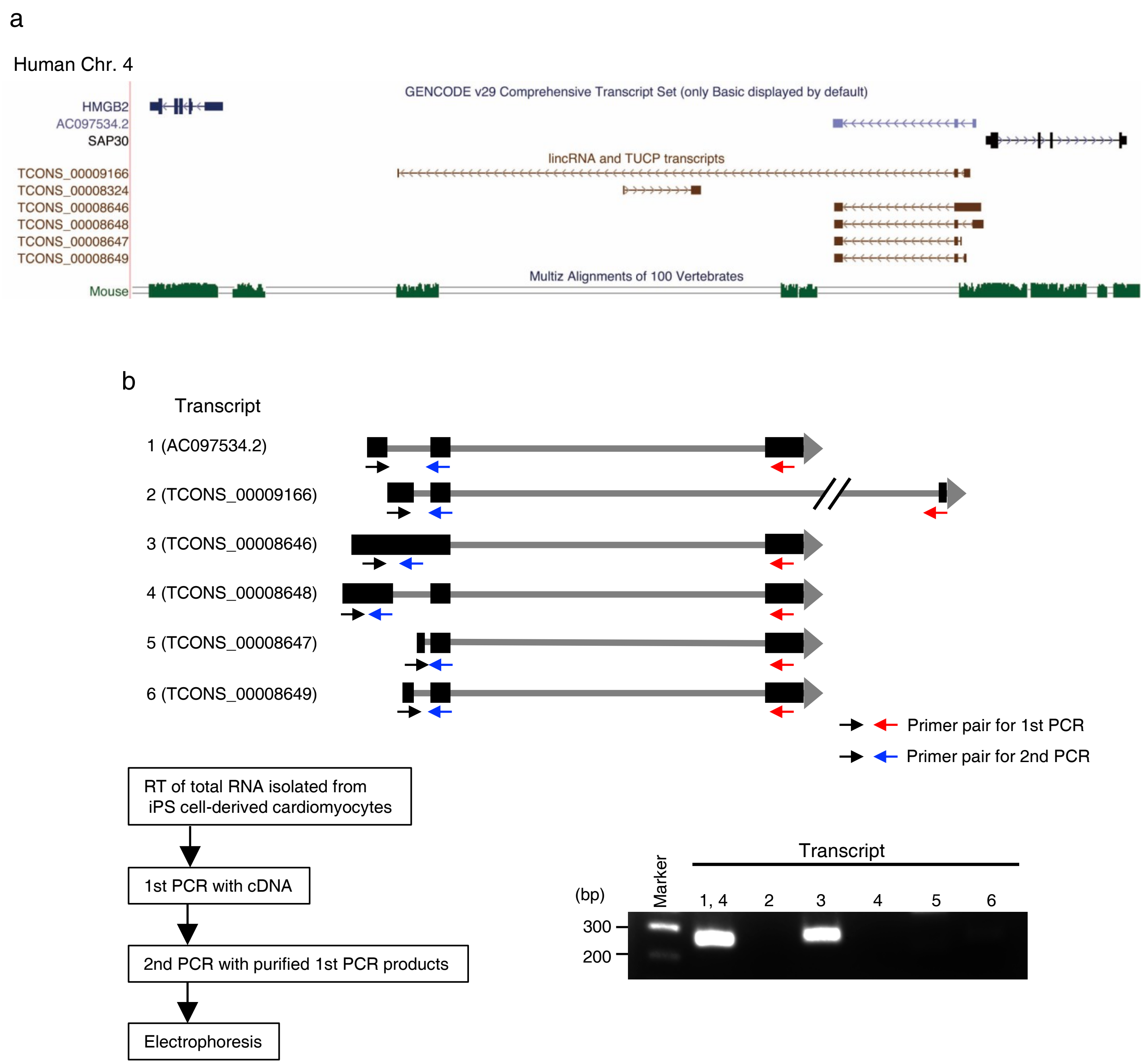

Supplementary Fig. 2 The intergenic region between human *HMGB2* and *SAP30* gives rise to several lincRNA transcripts.

a, UCSC genome browser snapshot showing *HMGB2* and *SAP30* loci on human chromosome 4 (GRCh38/hg38) and *lincRNA* transcripts and transcripts of uncertain coding potential (TUCPs) in that intergenic region. Green histogram indicates genomic sequences conserved in mice and humans. **b**, Schematic showing 6 human lincRNA transcripts (upper), a summary of semi-nested RT-PCR analysis of human lincRNA transcripts in human iPS cell-derived cardiomyocytes (lower left), and representative gel image of PCR products (lower right). Source data are provided as a Source Data file.

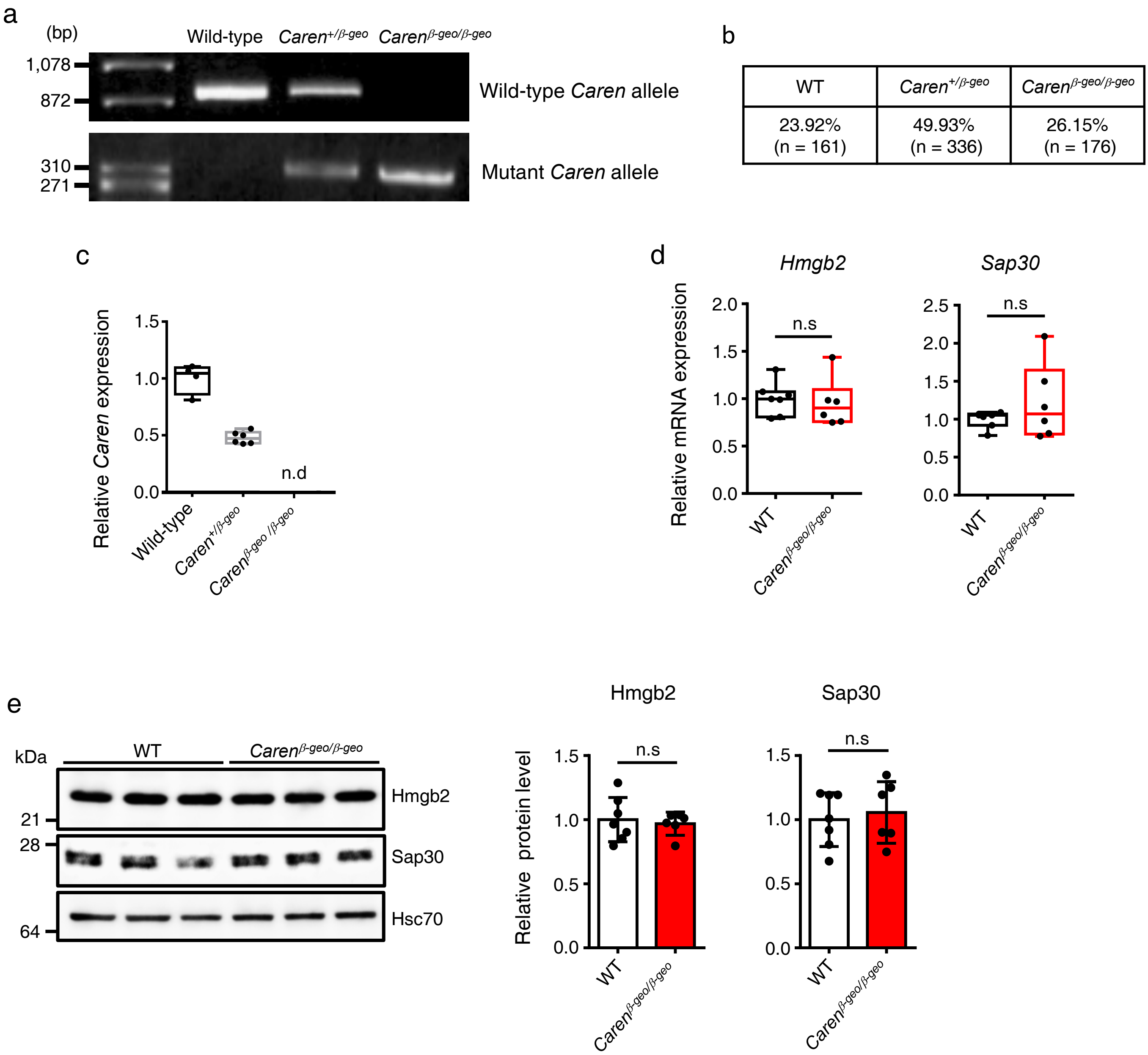

Supplementary Fig. 3 Expression of flanking genes is unchanged in the *Caren*-deficient heart.

a, Genotyping of WT, *Caren*$^{+/\beta\text{-geo}}$ and *Caren*$^{\beta\text{-geo}/\beta\text{-geo}}$ mice. **b**, Mendelian ratio of offspring of crosses of *Caren*$^{+/\beta\text{-geo}}$ mice at 6–8 weeks old. **c**, Relative *Caren* expression in heart tissues of indicated 12-week-old mice. WT levels were set to 1 (Wild type: n = 4, *Caren*$^{+/\beta\text{-geo}}$: n = 6, *Caren*$^{\beta\text{-geo}/\beta\text{-geo}}$: n = 4). **d**, Relative expression of the flanking genes *Hmgb2* (left) and *Sap30* (right) in hearts of *Caren*$^{\beta\text{-geo}/\beta\text{-geo}}$ or WT littermate mice (WT: n = 7, *Caren*$^{\beta\text{-geo}/\beta\text{-geo}}$: n = 6). **e**, Representative western blot (left) and quantitation (right) of Hmgb2 and Sap30 protein levels in heart tissues of *Caren*$^{\beta\text{-geo}/\beta\text{-geo}}$ or WT littermate mice (WT: n = 7, *Caren*$^{\beta\text{-geo}/\beta\text{-geo}}$: n = 6). Hsc70 served as a loading control. Protein intensity values

in sham-operated WT heart tissue were set to 1. Box plots for **c** and **d** present min to max, median and all points. For **e**, data show mean ± SD and all points. Statistical significance was determined by two-sided unpaired Student's *t* -test (**d** and **e**). n.s; not significant, between genotypes. n.d; not detected. Source data are provided as a Source Data file.

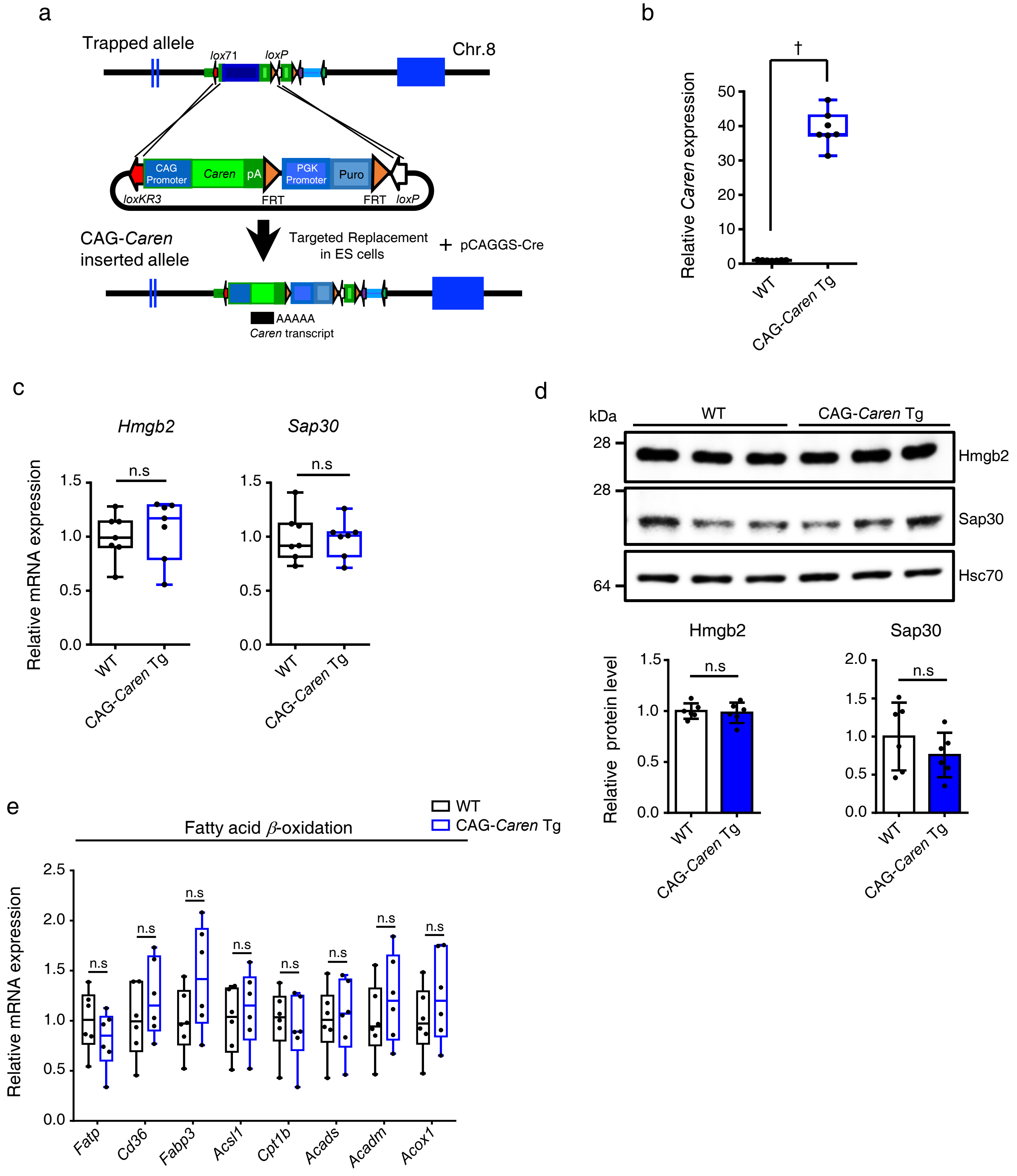

Supplementary Fig. 4 Expression of flanking genes is unchanged in the heart of CAG-*Caren* Tg mice.

a, Schematic showing generation of transgenic mice systemically overexpressing *Caren* (CAG-*Caren* Tg mice). **b**, Quantification of *Caren* expression in whole heart tissues of CAG-*Caren* Tg and littermate WT lines (n = 7 per group). Levels seen in WT mice were set to 1. **c**, Relative expression of *Hmgb2* (left) and *Sap30*

(right) in hearts from CAG-*Caren* Tg mice or littermate WT mice (n = 7 per group). **d**, Representative western blot (left) and quantitation (right) of Hmgb2 and Sap30 protein levels in heart tissues of WT mice or CAG-*Caren* Tg mice (n = 6 per group). Hsc70 served as a loading control. Protein intensity values of sham-operated WT heart tissue were set to 1. **e**, Relative expression of fatty acid β-oxidation-related transcripts in heart tissues of 12-week-old CAG-*Caren* Tg and WT littermate mice (n = 6 per group). WT values were set to 1. Box plots for **b**, **c**, and **e** present min to max, median, and all points. For **d**, data show mean ± SD and all points. Statistical significance was determined by two-sided unpaired Student's *t* -test (**b**–**e**). $^{\dagger}p < 0.0001$, n.s; not significant, between genotypes. Source data are provided as a Source Data file.

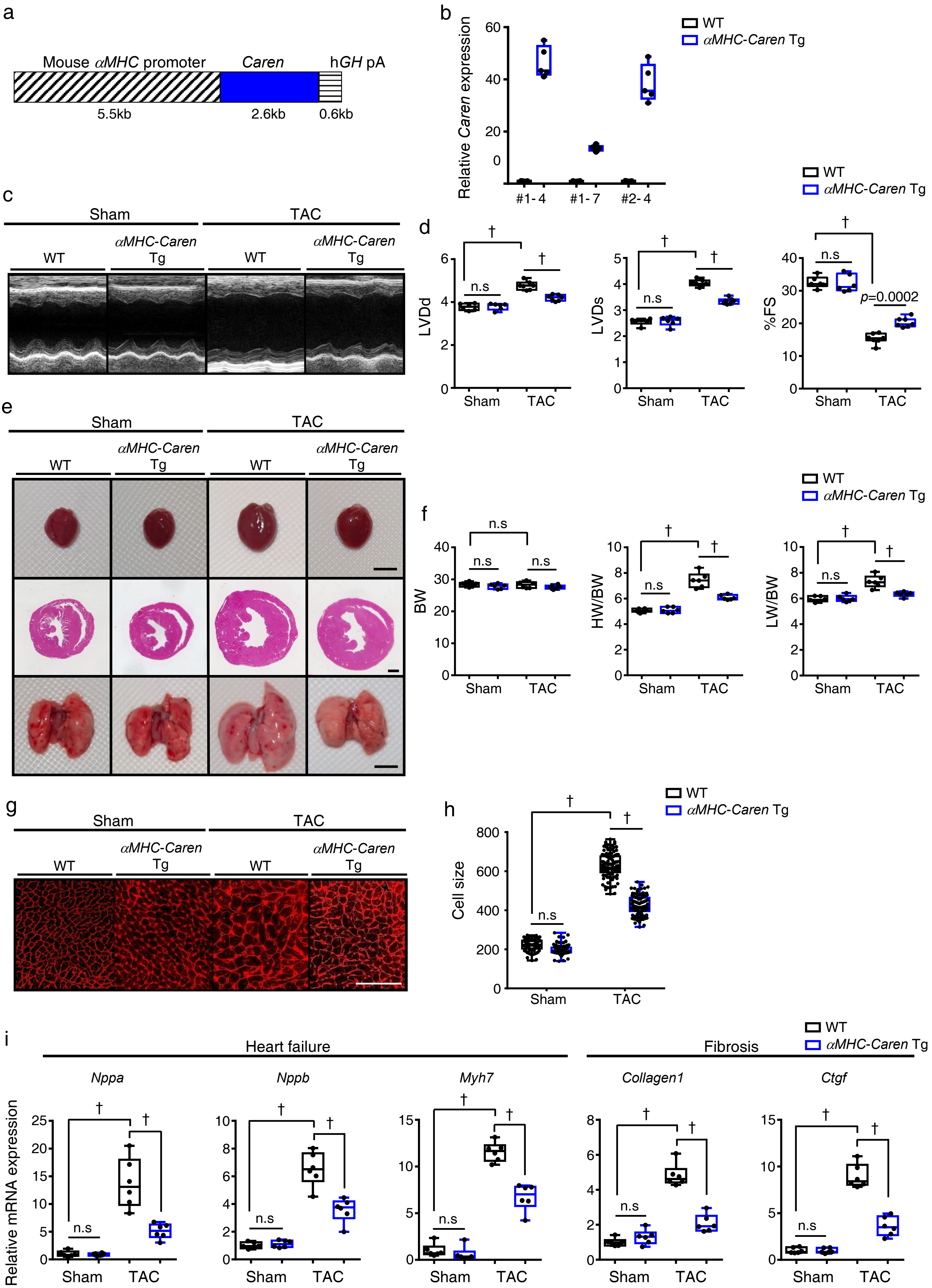
a
Mouse αMHC promoter
Caren
hGH pA
5.5kb
2.6kb
0.6kb
b
Relative Caren expression
WT
αMHC-Caren Tg
#1-4
#1-7
#2-4
c
Sham
TAC
WT
αMHC-Caren Tg
d
LVDd
LVDs
%FS
n.s
p=0.0002
e
f
BW
HW/BW
LW/BW
g
h
Cell size
i
Heart failure
Fibrosis
Nppa
Nppb
Myh7
Collagen1
Ctgf
Relative mRNA expression

Supplementary Fig. 5 *Caren* overexpression in mouse cardiomyocytes confers HF resistance.

a, Schematic showing transgene used to generate *αMHC-Caren* transgenic mice (*αMHC-Caren* Tg). h*GH* pA, human growth hormone poly A. **b**, Quantification of *Caren* transcript levels in hearts of Tg and WT littermate lines (WT littermate and Tg #1-7: n = 6 each, WT littermate and Tg #1-4: n = 5 each, WT littermate and Tg #2-4: n = 5 each). **c**, Shown are representative M-mode echocardiography recordings from *αMHC-Caren* Tg and WT littermate mice, 6 weeks after TAC or sham surgery. **d**, Left ventricular end-diastolic diameter (LVDd) (mm) (left), left ventricular end-systolic diameter (LVDs) (mm) (middle), and percent fractional shortening (%FS) (right) in indicated mice (WT sham and *αMHC-Caren* Tg sham: n = 6 per group, WT TAC and *αMHC-Caren* Tg TAC n = 7 per group). **e**, Gross appearance of whole heart (top row; scale bar, 5 mm), hematoxylin-eosin (HE)-stained sections of the heart mid-portion (middle row; scale bar, 1 mm), and gross appearance of whole lung (bottom row; scale bar, 5 mm). **f**, Body weight (BW) (g) (left), HW/BW ratio (mg/g) (middle), and lung weight per body weight ratio (LW/BW) (mg/g) (right) in indicated mice (WT sham and *αMHC-Caren* Tg sham: n = 6 per group, WT TAC and *αMHC-Caren* Tg TAC: n = 6 per group). **g**, Shown are representative left ventricle sections stained with wheat germ agglutinin (WGA) as an indicator of cardiomyocyte size (scale bar, 50 μm). **h**, Distribution sizes of myocardial cells (μm^2) in indicated mice (WT sham: n = 113 cells, *αMHC-Caren* Tg sham: n = 115 cells, WT TAC: n = 117 cells, *αMHC-Caren* Tg TAC: n =110 cells). **i**, Relative expression of genes associated with heart failure and fibrosis in hearts from indicated mice (n = 6 per group). Levels seen in sham-operated WT mice were set to 1. Box plots for **b**, **d**, **f**, **h**, and **i** present min to max, median and all points. Statistical significance was determined by one-way ANOVA with Sidak's post hoc test (**d**, **f**, **h**, and **i**). $^{\dagger}p < 0.0001$, n.s; not significant, between groups. Source data are provided as a Source Data file.

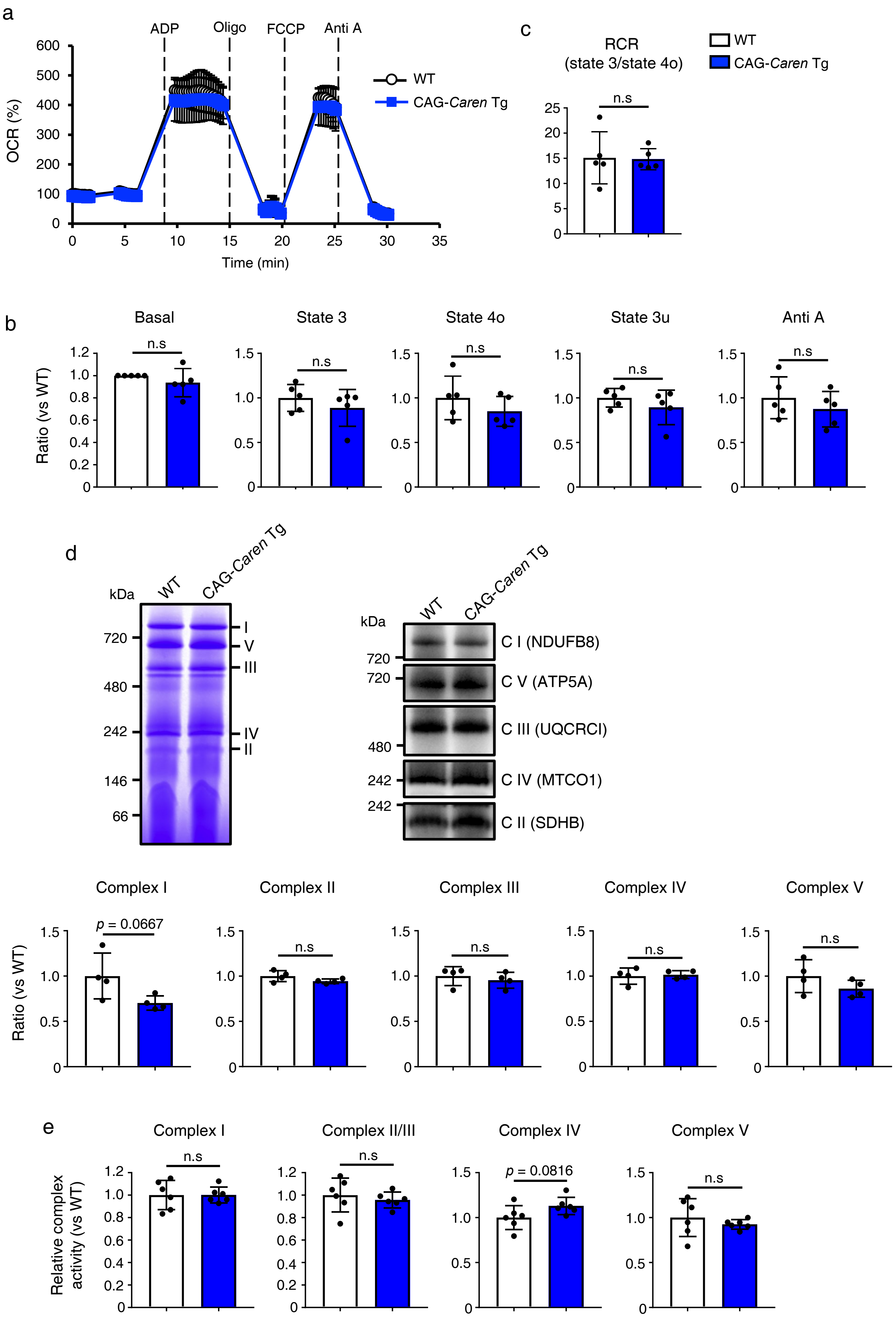
a
ADP
Oligo
FCCP
Anti A
OCR (%)
Time (min)
WT
CAG-Caren Tg
c
RCR
(state 3/state 4o)
n.s
b
Basal
State 3
State 4o
State 3u
Anti A
Ratio (vs WT)
d
kDa
CAG-Caren Tg
C I (NDUFB8)
C V (ATP5A)
C III (UQCRCI)
C IV (MTCO1)
C II (SDHB)
Complex I
Complex II
Complex III
Complex IV
Complex V
p = 0.0667
e
Complex II/III
p = 0.0816
Relative complex
activity (vs WT)

Supplementary Fig. 6 Mitochondrial respiratory function in *Caren*-overexpressing heart.

a, Oxygen consumption rate (OCR) of mitochondria isolated from heart tissues of CAG-*Caren* Tg and WT littermate mice (n = 5 per group). Basal OCR values seen in WT mice were set at 100%. **b**, Quantification of basal respiration, phosphorylating respiration in the presence of ADP (State 3), resting respiration in the presence of oligomycin (State 4o), maximal uncoupling respiration in the presence of FCCP (State 3u), and respiration in the presence of antimycin A (Anti A) (n = 5 per group). WT values were set at 1. **c**, Respiratory control ratio (RCR, state 3/state 4o) of isolated heart mitochondria from CAG-*Caren* Tg and WT littermate mice (n = 5 per group). **d**, Representative BN-PAGE (top left) and immunoblotting (top right) analyses of heart mitochondria isolated from CAG-*Caren* Tg and WT littermate mice, and quantification of levels of indicated mitochondrial respiratory chain complexes (bottom) (n = 4 per group). Levels in WT mice were set at 1. **e**, Relative activities of indicated mitochondrial respiratory chain complexes in isolated heart mitochondria from CAG-*Caren* Tg and WT littermate mice (n = 6 per group). WT values were set at 1. Data show mean ± SD for **a**. For **b**–**e**, data show mean ± SD and all points. Statistical significance was determined by two-sided unpaired Student's *t*-test (**b**–**e**). n.s; not significant, between genotypes. Source data are provided as a Source Data file.

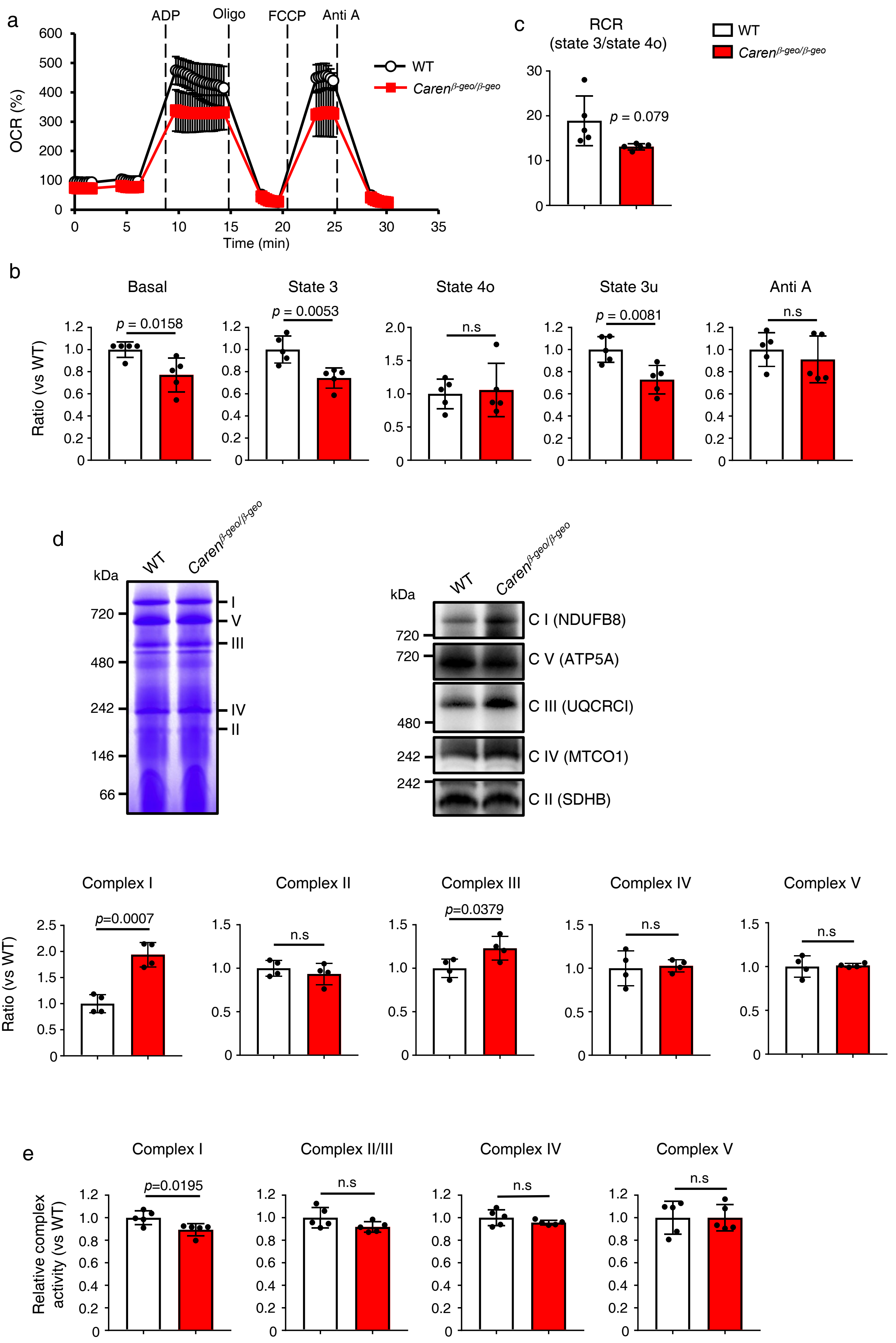

a
ADP
Oligo
FCCP
Anti A
OCR (%)
Time (min)
WT
Caren$^{β-geo/β-geo}$
b
Basal
State 3
State 4o
State 3u
Anti A
Ratio (vs WT)
p = 0.0158
p = 0.0053
n.s
p = 0.0081
n.s
c
RCR
(state 3/state 4o)
p = 0.079
WT
Caren$^{β-geo/β-geo}$
d
WT
Caren$^{β-geo/β-geo}$
kDa
720
480
242
146
66
I
V
III
IV
II
C I (NDUFB8)
C V (ATP5A)
C III (UQCRCI)
C IV (MTCO1)
C II (SDHB)
Complex I
Complex II
Complex III
Complex IV
Complex V
Ratio (vs WT)
p=0.0007
n.s
p=0.0379
n.s
n.s
e
Complex I
Complex II/III
Complex IV
Complex V
Relative complex activity (vs WT)
p=0.0195
n.s
n.s
n.s

Supplementary Fig. 7 Mitochondrial respiratory function in *Caren*-deficient heart.

a, OCR of mitochondria isolated from heart tissues of $Caren^{\beta\text{-}geo/\beta\text{-}geo}$ and WT littermate mice (n = 5 per group). Basal OCR values seen in WT mice were set at 100%. **b**, Quantification of basal, State 3, State 4o, and State 3u respiration and respiration in the presence of antimycin A (Anti A) (n = 5 per group). WT values were set at 1. **c**, RCR (state 3/state 4o) of heart mitochondria isolated from CAG-*Caren* Tg and WT littermate mice (n = 5 per group). **d**, Representative BN-PAGE (top left) and immunoblotting (top right) analyses of heart mitochondria isolated from $Caren^{\beta\text{-}geo/\beta\text{-}geo}$ and WT littermate mice, and quantification of levels of indicated mitochondrial respiratory chain complexes (bottom) (n = 4 per group). Levels in WT mice were set at 1. **e**, Relative activities of indicated mitochondrial respiratory chain complexes in heart mitochondria isolated from $Caren^{\beta\text{-}geo/\beta\text{-}geo}$ and WT littermate mice (n = 5 per group). WT values were set at 1. Data show mean ± SD for **a**. For **b**–**e**, data show mean ± SD and all points. Statistical significance was determined by two-sided unpaired Student's *t* -test (**b**–**e**). $^{\dagger}p < 0.0001$, n.s; not significant, between genotypes. Source data are provided as a Source Data file.

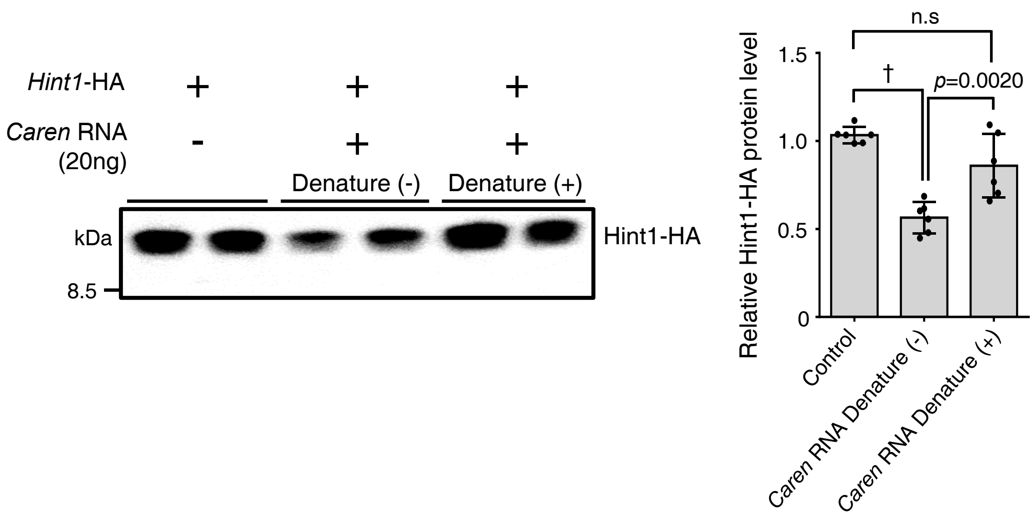

Supplementary Fig. 8 Normal secondary structure is required for *Caren* to suppress Hint1 translation.

Representative western blot (left) and quantitation (right) of *in vitro* translated *Hint1* mRNA with native and denature forms of 20 ng *Caren* RNA (n = 6 per group). Data show mean ± SD and all points. Statistical significance was determined by one-way ANOVA with Sidak's post hoc test. †$p < 0.0001$, n.s; not significant, between groups.

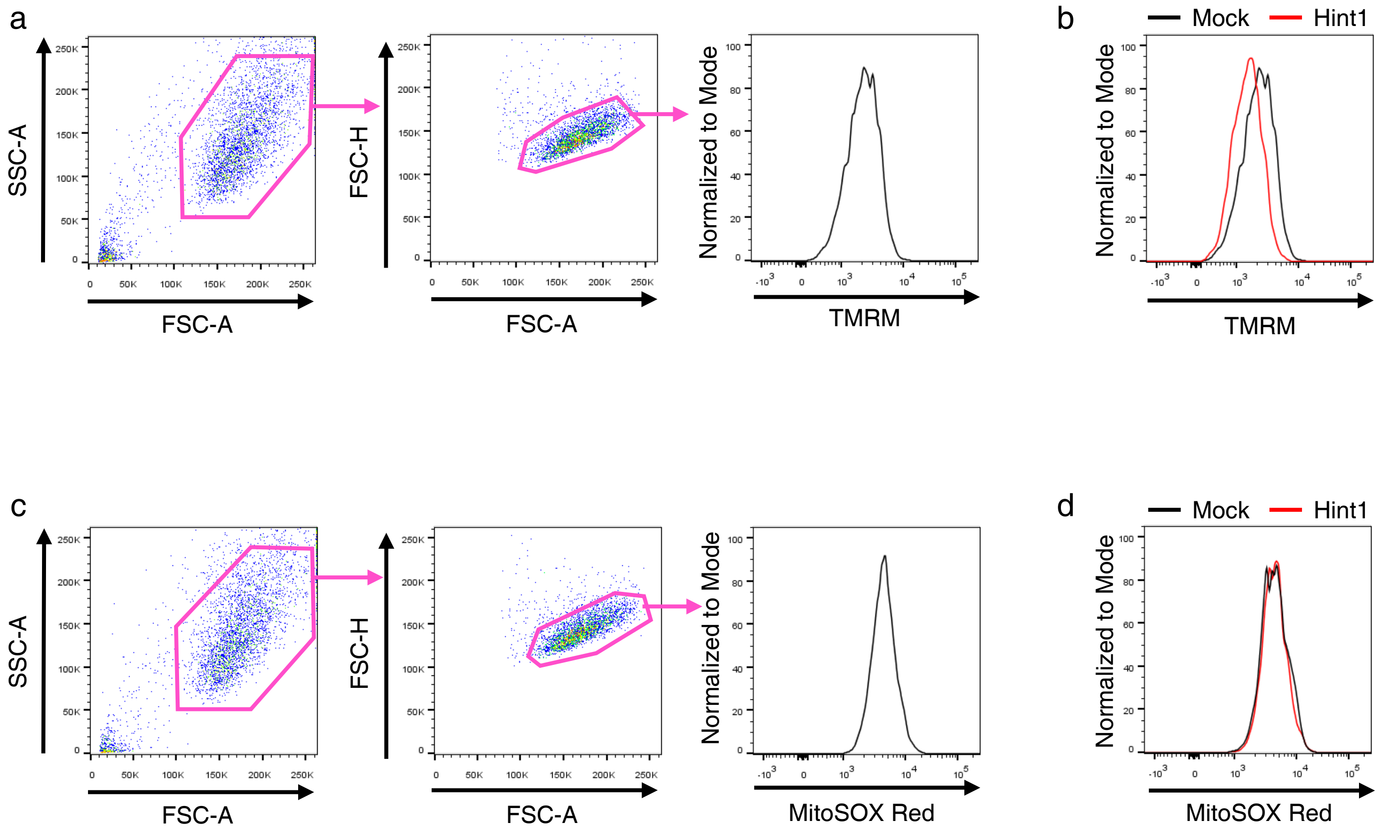

Supplementary Fig. 9 Gating strategies used to determine mitochondrial membrane potential and ROS levels in H9c2-Hint1 cells.

a, **b**, Gating strategy used to analyze mitochondrial membrane potential with TMRM in H9c2 cells (**a**) and representative histogram of TMRM staining in H9c2-mock and H9c2-Hint1 cells (**b**) (Fig. 6d). **c**, **d**, Gating strategy used to analyze mitochondrial ROS levels with MitoSOX Red in H9c2 cells (**c**) and representative histogram of MitoSOX Red staining in H9c2-mock and H9c2-Hint1 cells (**d**) (Fig. 6e).

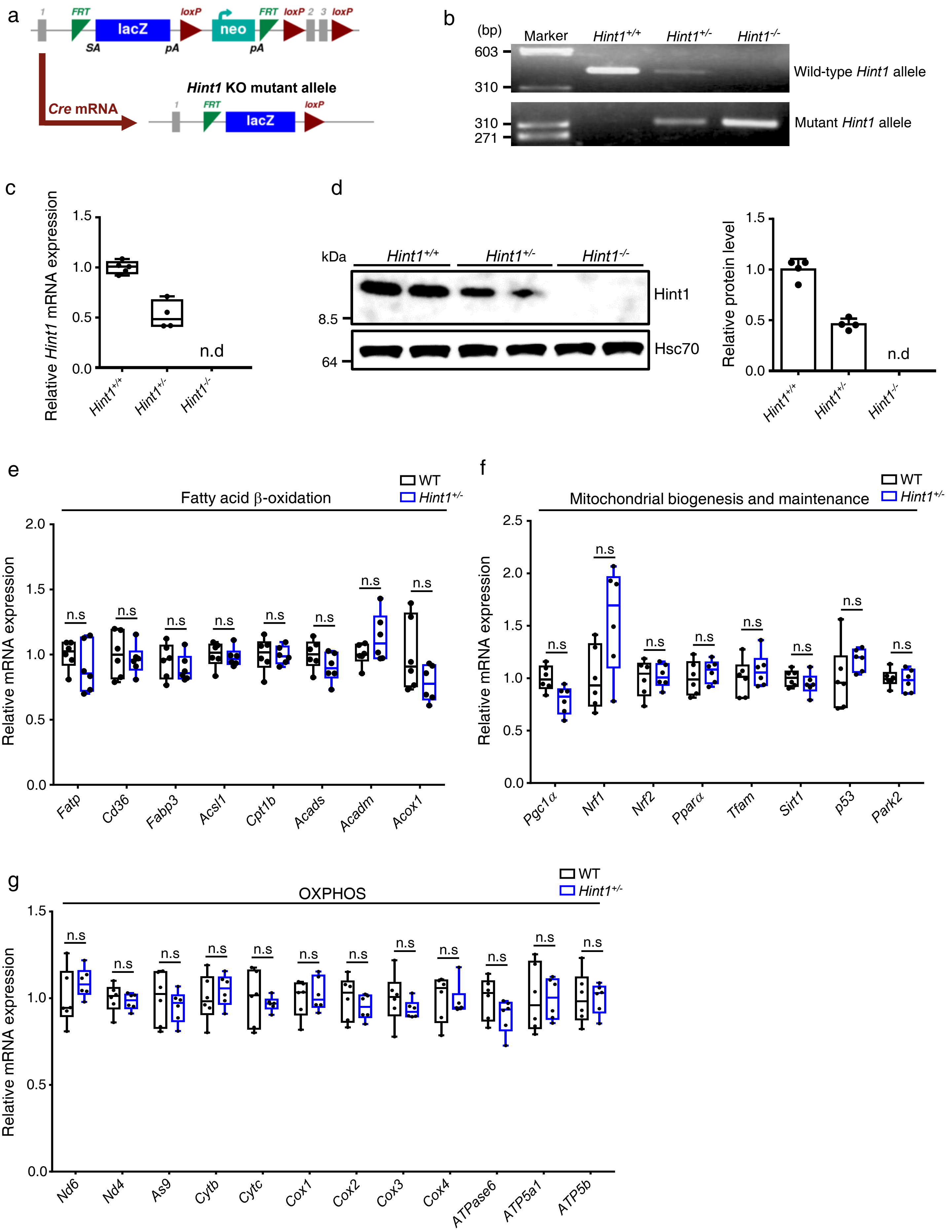

a
FRT
lacZ
loxP
neo
FRT
loxP
loxP
SA
pA
pA
Hint1 KO mutant allele
Cre mRNA
FRT
lacZ
loxP
b
(bp)
Marker
Hint1+/+
Hint1+/-
Hint1-/-
603
310
310
271
Wild-type Hint1 allele
Mutant Hint1 allele
c
Relative Hint1 mRNA expression
n.d
d
kDa
Hint1
Hsc70
8.5
64
Relative protein level
n.d
e
Fatty acid β-oxidation
WT
Hint1+/-
Relative mRNA expression
Fatp
Cd36
Fabp3
Acsl1
Cpt1b
Acads
Acadm
Acox1
f
Mitochondrial biogenesis and maintenance
Pgc1α
Nrf1
Nrf2
Pparα
Tfam
Sirt1
p53
Park2
g
OXPHOS
Nd6
Nd4
As9
Cytb
Cytc
Cox1
Cox2
Cox3
Cox4
ATPase6
ATP5a1
ATP5b

Supplementary Fig. 10 Establishment of *Hint1* knockout mice.

a, Schematic showing the *Hint1* mutant allele. The cassette (top) is composed of a short flippase recombination enzyme (Flp)-recognition target (FRT), a lacZ reporter, the neomycin resistance gene and a Cre recombinase recognition target (loxP). (Bottom) *Hint1* knockout allele with *lacZ* encoding the β-gal reporter, as generated by Cre recombinase. **b**, Genotyping of *Hint1*$^{+/+}$, *Hint1*$^{+/-}$, and *Hint1*$^{-/-}$ mice. **c**, Quantification of *Caren* expression in heart tissues of 12-week-old *Hint1*$^{+/+}$, *Hint1*$^{+/-}$ and *Hint1*$^{-/-}$ mice. (*Hint1*$^{+/+}$: n = 5, *Hint1*$^{+/-}$: n = 5, *Hint1*$^{-/-}$: n = 4). **d**, Representative western blot (left) and quantitation (right) of Hint1 protein levels in heart tissues of 12-week-old *Hint1*$^{+/+}$, *Hint1*$^{+/-}$, and *Hint1*$^{-/-}$ mice (n = 4 per group). Hsc70 served as a loading control. Protein intensity values in the *Hint1*$^{+/+}$ group were set to 1. **e**–**g**, Relative expression of genes associated with fatty acid β-oxidation (**e**), mitochondrial biogenesis and maintenance (**f**), or OXPHOS (**g**) in heart tissues of 12-week-old *Hint1*$^{+/-}$ and WT littermate mice (n = 6 per group). WT values were set to 1. Box plots for **c** and **e**–**g** present min to max, median and all points. For **d**, data show mean ± SD and all points. Statistical significance was determined by two-sided unpaired Student's *t*-test (**e**–**g**). n.s; not significant, between genotypes. n.d; not detected. Source data are provided as a Source Data file.

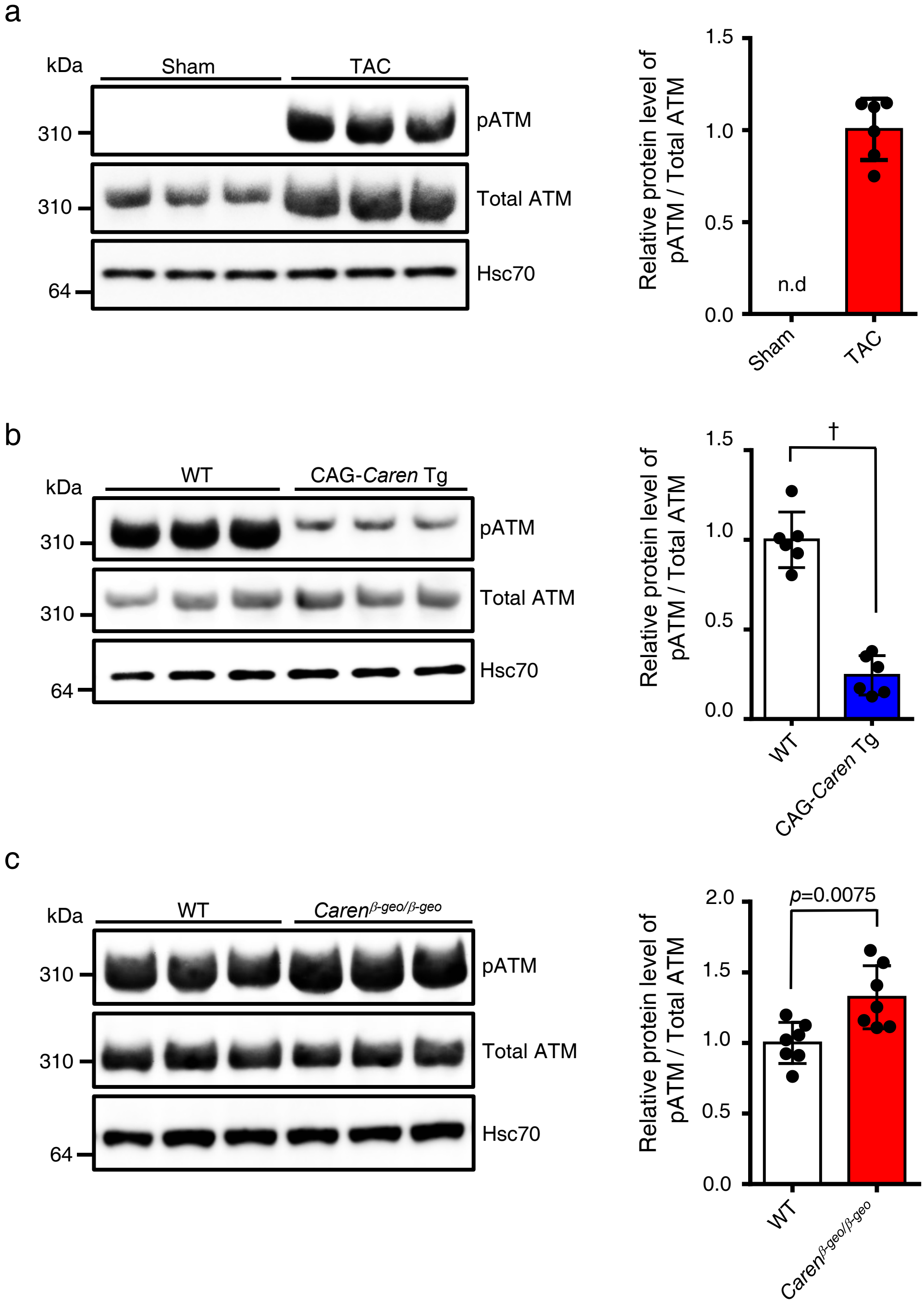

Supplementary Fig. 11 ATM phosphorylation levels in *Caren*-overexpressing and -deficient heart.

a–**c**, Representative western blot (left) and pATM/total ATM ratio (right) in heart tissues of WT mice, 6 weeks after TAC or sham surgery (n = 6 per group) (**a**), CAG-*Caren* Tg and WT mice, 6 weeks after TAC (n = 6 per group) (**b**), *Caren*$^{\beta\text{-}geo/\beta\text{-}geo}$ and WT mice, 4 weeks after TAC (n = 7 per group) (**c**). Hsc70 served as a loading control. Values derived from sham-operated mouse heart tissue or WT mouse heart tissue were set to 1. Data show mean ± SD and all points. Statistical significance was determined by two-sided unpaired Student's *t*-test. $^{\dagger}p < 0.0001$. n.d; not detected. Source data are provided as a Source Data file.

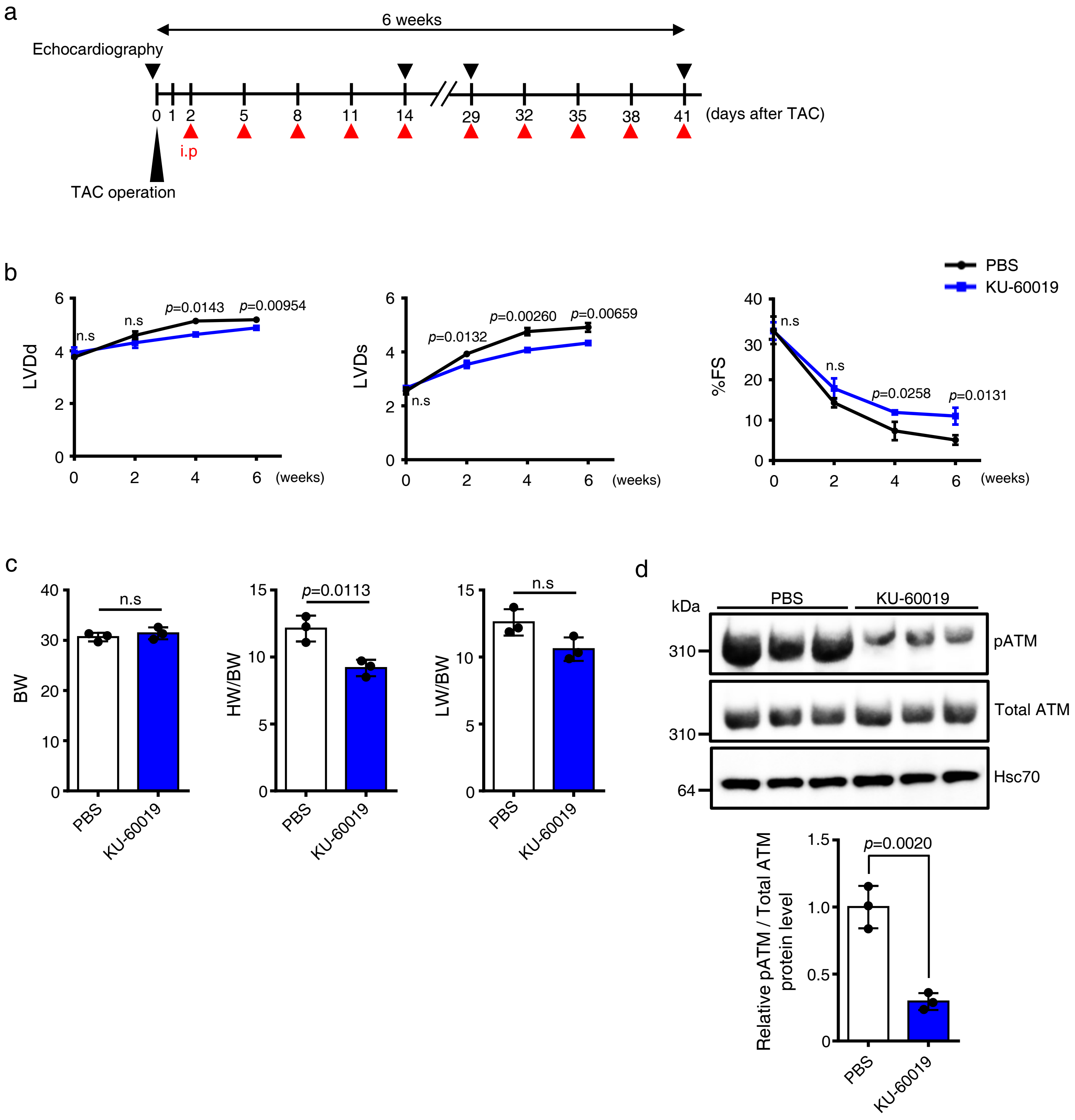

Supplementary Fig. 12 ATM inhibition suppresses HF progression in *Caren*-deficient mice subjected to pressure overload.

a, Schematic showing experimental protocol for ATM inhibitor (KU-60019) treatment. **b**, Left ventricular end-diastolic diameter (LVDd) (mm) (left), left ventricular end-systolic diameter (LVDs) (mm) (middle), and percent fractional shortening (%FS) (right) in TAC-operated *Caren* $^{\beta\text{-}geo/\beta\text{-}geo}$ mice administered KU-60019 or PBS (n = 3 per group). **c**, Body weight (BW) (g) (left), HW/BW ratio

(mg/g) (middle), and lung weight per body weight ratio (LW/BW) (mg/g) (right) in KU-60019- or vehicle (PBS)-treated *Caren* $^{\beta\text{-}geo/\beta\text{-}geo}$ mice, after 6 weeks TAC surgery (n = 3 per group). **d**, Representative western blot (top) and pATM/total ATM ratio (bottom) in heart tissues of KU-60019- or vehicle (PBS)-treated *Caren* $^{\beta\text{-}geo/\beta\text{-}geo}$ mice, 6 weeks after TAC surgery (n = 3 per group). Hsc70 served as a loading control. Values derived from vehicle-treated mouse heart tissue were set to 1. Data show mean ± SD and all points for **c** and **d**. For **b**, data show mean ± SD. Statistical significance was determined by two-sided unpaired Student's *t* - test (**b**–**d**). n.s; not significant, between groups. Source data are provided as a Source Data file.

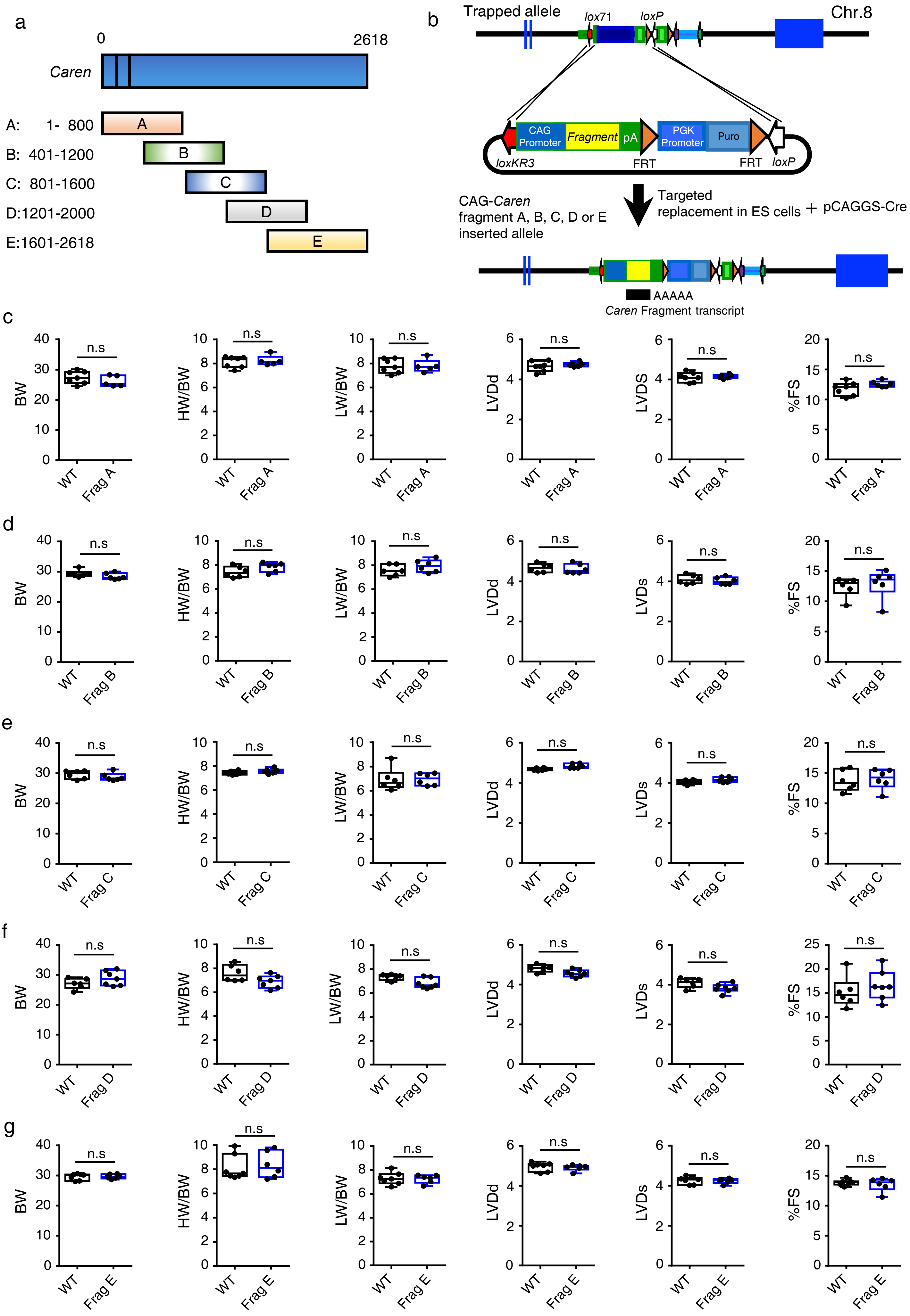
a
0
2618
Caren
A: 1- 800
B: 401-1200
C: 801-1600
D:1201-2000
E:1601-2618
b
Trapped allele
lox71
loxP
Chr.8
CAG Promoter
Fragment
pA
PGK Promoter
Puro
loxKR3
FRT
FRT
loxP
CAG-Caren fragment A, B, C, D or E inserted allele
Targeted replacement in ES cells
+ pCAGGS-Cre
AAAAA
Caren Fragment transcript
c
d
e
f
g
BW
HW/BW
LW/BW
LVDd
LVDS
LVDs
%FS
n.s
WT
Frag A
Frag B
Frag C
Frag D
Frag E

Supplementary Fig. 13 Analysis of transgenic mice overexpressing various *Caren* fragments.

a, Schematic showing sequential overlapping fragments of *Caren* sequence used in functional analysis. **b**, Schematic showing construct used to generate transgenic mice harboring various *Caren* fragments (CAG-*Caren* fragment A, B, C, D, and E Tg). **c**–**g**, Indicated parameters assessed in Tg mice harboring various CAG-*Caren* fragments and in WT littermate mice 6 weeks after TAC or sham surgery (WT: n = 6–7, Tg: n = 5–7). Box plots for **c**–**g** present min to max, median and all points. Statistical significance was determined by two-sided unpaired Student's *t* -test (**c**–**g**). n.s; not significant, between genotypes. Source data including exact 'n' each group, are provided as a Source Data file.

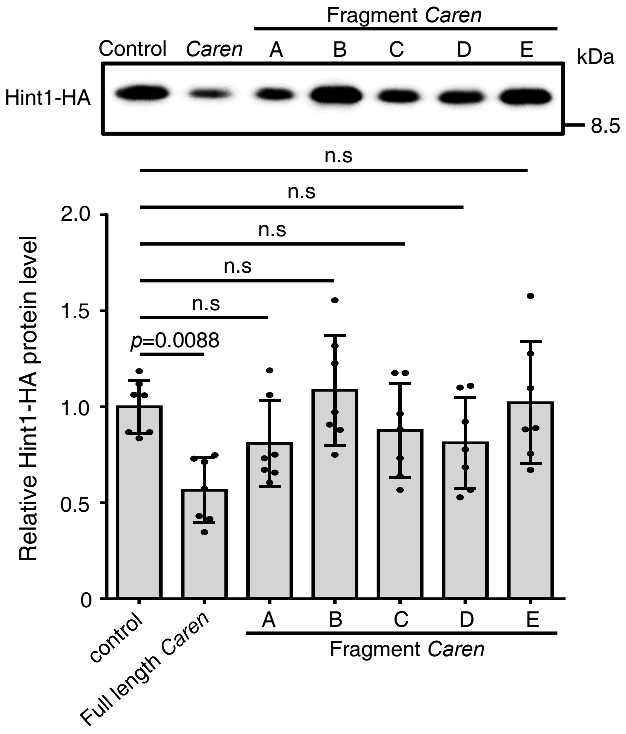

Supplementary Fig. 14 *Caren* lncRNA structural integrity is important for the suppression of Hint1 translation.

Representative western blot (top) and quantitation (bottom) of *in vitro* translated *Hint1* mRNA with *Caren* fragment A, B, C, D, or E (n = 7 per group). Data show mean ± SD and all points. Statistical significance was determined by one-way ANOVA with Sidak's post hoc test. n.s; not significant, between groups. Source data are provided as a Source Data file.

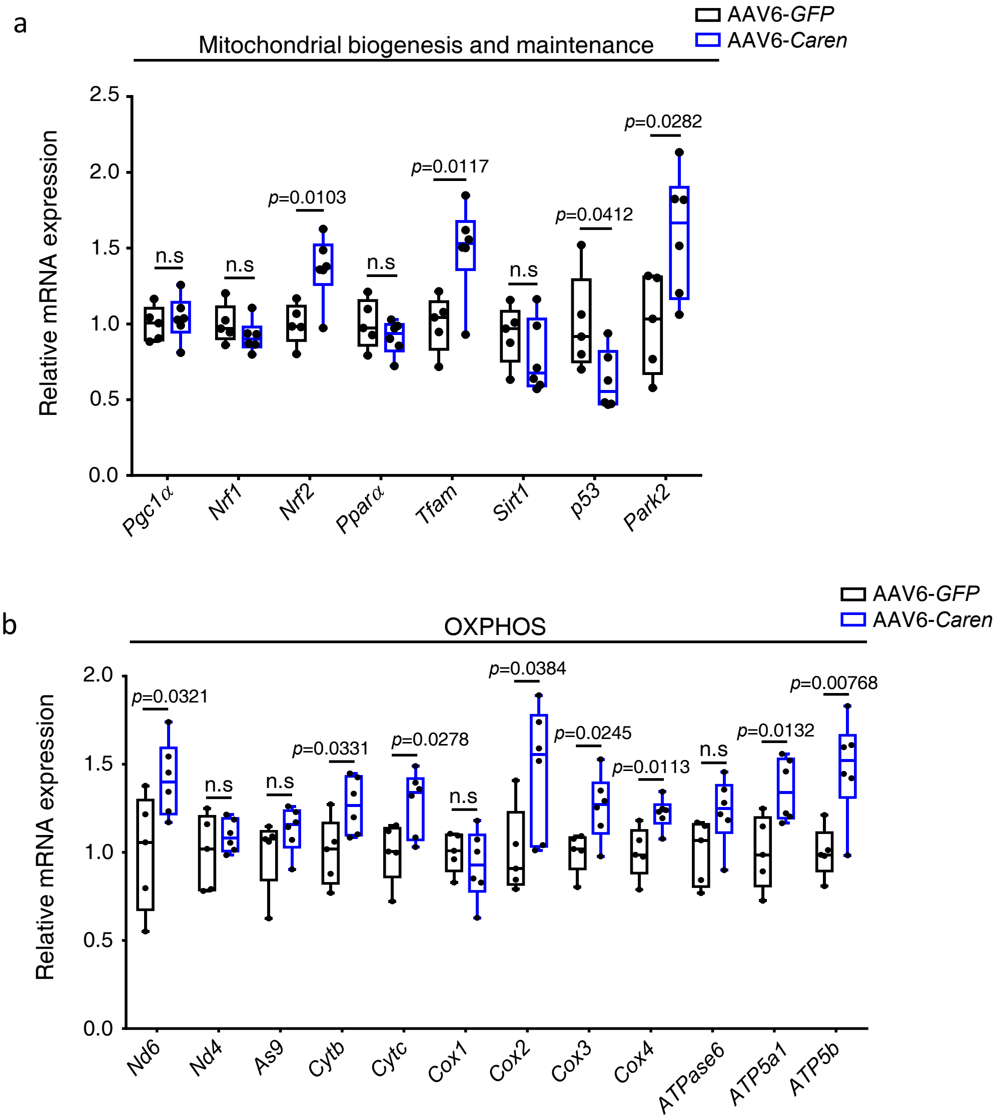

Supplementary Fig. 15 Intravenous injection of AAV6-*Caren* increases the expression levels of genes functioning in mitochondrial biogenesis and OXPHOS in heart.

a, **b** Relative expression of genes associated with mitochondrial biogenesis and maintenance (**a**) or OXPHOS (**b**) in heart tissues of WT mice injected with 1 × 10^{11} vg per mouse of AAV6-*GFP* or AAV6-*Caren*, 1 week after injection (AAV6-*GFP*: n = 5, AAV6-*Caren*: n = 6). Levels seen in the AAV6-*GFP* group were set to 1. Box plots present min to max, median and all points. Statistical significance was determined by two-sided unpaired Student's *t* -test. n.s; not significant, between groups. Source data are provided as a Source Data file.

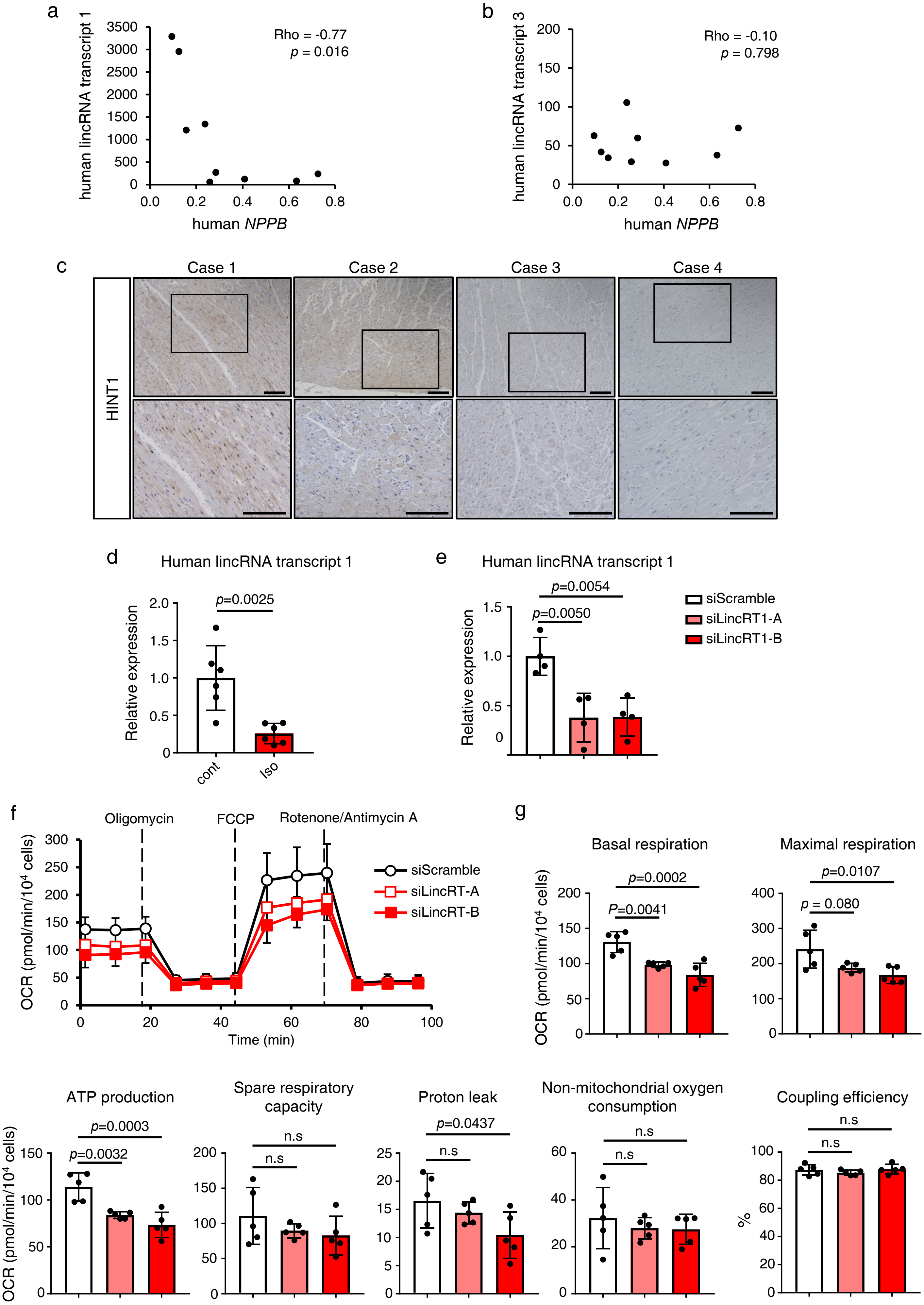

a
human lincRNA transcript 1
human NPPB
Rho = -0.77
p = 0.016
b
human lincRNA transcript 3
human NPPB
Rho = -0.10
p = 0.798
c
Case 1
Case 2
Case 3
Case 4
HINT1
d
Human lincRNA transcript 1
p=0.0025
Relative expression
cont
Iso
e
Human lincRNA transcript 1
p=0.0054
p=0.0050
Relative expression
siScramble
siLincRT1-A
siLincRT1-B
f
Oligomycin
FCCP
Rotenone/Antimycin A
OCR (pmol/min/10⁴ cells)
Time (min)
siScramble
siLincRT-A
siLincRT-B
g
Basal respiration
p=0.0002
P=0.0041
Maximal respiration
p=0.0107
p = 0.080
OCR (pmol/min/10⁴ cells)
ATP production
p=0.0003
p=0.0032
Spare respiratory capacity
n.s
n.s
Proton leak
p=0.0437
n.s
Non-mitochondrial oxygen consumption
n.s
n.s
Coupling efficiency
n.s
n.s
%

Supplementary Fig. 16 Analysis of human lincRNA transcripts in human heart tissues and cardiomyocytes.

a, **b**, Scatter plots showing correlation between human lncRNA transcripts and *NPPB* transcripts. Expression levels of human lincRNA transcripts 1 (**a**) and 3 (**b**) relative to *NPPB* (n = 9). Shown is Spearman's correlation coefficient r(ρ). **c**, Immunohistochemical staining for HINT1 in heart tissues from cases showing low levels of lincRNA transcript 1 expression and high levels of *NPPB* expression (Case 1: patient with amyotrophic lateral sclerosis; Case 2: patient with intrahepatic cholangiocarcinoma) or with high levels of lincRNA transcript 1 transcripts and low levels of *NPPB* (Case 3: patient with interstitial lung disease due to polymyositis; Case 4: patient with hypertrophic obstructive cardiomyopathy). Lower panels show magnified images of boxed regions in upper panels. Scale bars: 100 μm. **d**, Relative expression of human lincRNA transcript 1 in isoproterenol (Iso)-treated or untreated human iPSC-CMs (n = 6 per group). Levels seen in untreated cells were set at 1. **e**, Relative expression of human lincRNA transcript 1 in human iPSC-CMs transfected with control siRNA (siScramble) or either of two human lincRNA transcript 1-specific siRNAs (siLincRT1-A and siLincRT1-B) (n = 4 per group). Levels seen in control cells were set at 1. **f**, Mitochondrial oxygen consumption rate (OCR) in control and lincRNA transcript 1-knockdown human iPSC-CMs (n = 5 biological replicates per group). **g**, Quantification of basal respiration, maximal respiration, ATP production, spare respiratory capacity, proton leak, non-mitochondrial respiration, and coupling efficiency in control and lincRNA transcript 1-knockdown human iPSC-CMs (n = 5 biological replicates per group). Data show mean ± SD and all points for **d**, **e**, and **g**. For **f**, data show mean ± SD. Statistical significance was determined by two-sided unpaired Student's *t* -test (**d**) or one-way ANOVA with Sidak's post hoc test (**e** and **g**). $^{\dagger}p < 0.0001$, n.s; not significant, between groups. Source data are provided as a Source Data file.

Supplementary Table 1. Individuals' cause of death and HINT1 expression based on 9 autopsy cases.

Case No.	Cause of death	HINT1 expression in heart tissue
1	Amyotrophic lateral sclerosis	(+++)
2	Intrahepatic cholangiocarcinoma	(+++)
3	Interstitial lung disease due to polymyositis	(+)
4	Hypertrophic obstructive cardiomyopathy	(+)
5	Peritoneal carcinomatosis	(++)
6	Lung cancer	(+)
7	Septic shock due to arteriosclerosis obliterans	(+++)
8	T cell lymphoma	(++)
9	Pancreatitis	(++)

Supplementary Table 2. Primer pairs used for quantitative RT-PCR

Gene		Sequences
Mouse		
Rps18	Forward	TTCTGGCCAACGGTCTAGACAAC
	Reverse	CCAGTGGTCTTGGTGTGCTGA
Nppa	Forward	GAGAGACGGCAGTGCTTCTAGGC
	Reverse	CGTGACACACCACAAGGGCTTAGG
Nppb	Forward	AGGCGAGACAAGGGAGAACA
	Reverse	GGAGATCCATGCCGCAGA
Myh6	Forward	CGCAATGCAGAGTCGGTGA
	Reverse	TCCTGCAGCCGCATTAAGTTC
Myh7	Forward	CGGACCTTGGAAGACCAGAT
	Reverse	GACAGCTCCCCATTCTCTGT
Collagen1	Forward	GAGCGGAGAGTACTGGATCGA
	Reverse	CTGACCTGTCTCCATGTTGCA
Ctgf	Forward	CAAAGCAGCTGCAAATACCA
	Reverse	GGCCAAATGTGTCTTCCAGT
Sirt1	Forward	TCCTCACTAATGGCTTTCATTCCTG
	Reverse	GTGCCAATCATGAGATGTTGCTG
Nrf1	Forward	TTGCCCAAGTGAATTACTCTGCTG
	Reverse	TGCAGGACAGTCTGAGCCATC
Nrf2	Forward	TTGGCAGAGACATTCCCATTTGTA
	Reverse	AGTCATGGCTGCCTCCAGAGA
Pparα	Forward	ACGCTCCCGACCCATCTTTAG
	Reverse	TCCATAAATCGGCACCAGGAA
Pgc-1α	Forward	CCGTAAATCTGCGGGATGATG
	Reverse	CAGTTTCGTTCGACCTGCGTAA
Tfam	Forward	TGAAGCTTGTAAATGAGGCTTGGA
	Reverse	CGGATCGTTTCACACTTCGAC
Trp53(P53)	Forward	GCCGATAGGTCGTTTCTTCC

	Reverse	ATCCGACTGTGACTCCTCCA
Park2	Forward	AGGACACGTCGGTAGCTTTG
	Reverse	CACTGGAAGACCAGGACAGG
Nd6	Forward	AGGTGAAGGCTTTAATGCTAACCC
	Reverse	GGTCGCAGTTGAATGCTGTGT
Nd4	Forward	CATCACTCCTATTCTGCCTAGCAA
	Reverse	TCCTCGGGCCATGATTATAGTAC
As9	Forward	CCCGGGCCAGCTTACCT
	Reverse	GCTGCACTGCTTTCCTGATAGA
Cyt.b	Forward	GCCACCTTGACCCGATTCT
	Reverse	TTGCTAGGGCCGCGATAAT
Cyt.c	Forward	GGCTGCTGGATTCTCTTACACA
	Reverse	CCAAATACTCCATCAGGGTATCCT
Cox1	Forward	TTTTCAGGCTTCACCCTAGATGA
	Reverse	GAAGAATGTTATGTTTACTCCTACGAATATG
Cox2	Forward	CCATCCCAGGCCGACTAAA
	Reverse	TTTCAGAGCATTGGCCATAGAA
Cox3	Forward	CGGAAGTATTTTTCTTTGCAGGAT
	Reverse	CAGCAGCCTCCTAGATCATGTG
Cox4	Forward	TGCAGACCAAGCGAATGCT
	Reverse	TAGTCCCACTTGGCGGAGAA
ATP5a1	Forward	ATGTGTCCGCTTACATTCCAACAA
	Reverse	GATCCGACACGGGACACAGA
ATP5b	Forward	ACATGGGCACAATGCAGGAA
	Reverse	GTCAGGTCATCAGCAGGCACA
ATPase6	Forward	GGCTCCCGACACAAACTAAAAAG
	Reverse	TGGAATTAGTGAAATTGGAGTTCCT
Fatp	Forward	GCAGCATTGCCAACATGGAC
	Reverse	GTGTCCTCATTGACCTTGACCAGA
Cd36	Forward	GATGGCCTTACTTGGGATTGGA

	Reverse	GGCTTTACCAAAGATGTAGCCAGTG
Fabp3	Forward	TGGCTAGCATGACCAAGCCTACTAC
	Reverse	GTTCCACTTCTGCACATGGATGA
Acsl1	Forward	TTTGCCTGCAGCGAGTGTG
	Reverse	GCCCTCGACTATCCCTATGGTAAGA
Cpt1α	Forward	GCCATGATGGACCCCACAAC
	Reverse	CCAGATACTTGGACACCACATAGAGG
Cpt1β	Forward	GAGACAGGACACTGTGTGGGTGA
	Reverse	AGTGCCTTGGCTACTTGGTACGAG
Acads	Forward	AAGTTTGGATCCGCACAGCAG
	Reverse	CAAGCTTTGGTGCCGTTGAG
Acadm	Forward	CGAGTATGTTATCAACGGCCAGAA
	Reverse	GCGGGTACTTTAGGATCTGGGTTAG
Acox1	Forward	AAGATGGATCCTAAGCCAGCTGAA
	Reverse	CAGCTTACCACAAAGCCAGCTACTC
Hmgb2	Forward	GTACATGCAATCCCAGAGTGGAA
	Reverse	CATTGAAGCATGTACTTTGGTGGTG
Sap30	Forward	CTTCAAGCTTCCAACCAGACCAG
	Reverse	ATGGGCTAGTGTGAACAGCATCTC
Hint1	Forward	AAATCCCCGCCAAGATCATC
	Reverse	GGGGAAATGTCATGAAAAGC
Human		
LincRNA	Forward	GACTTCTCAACTGTGACAGTTTG
transcript 1	Reverse	CGCGTCTGTAATCCCAGCTA
RPS18	Forward	TTTGCGAGTACTCAACACCAACATC
	Reverse	GAGCATATCTTCGGCCCACAC

Supplementary Table 3. Primer pairs used for quantification of mitochondrial DNA content

Gene		Sequences
CytB	Forward	GCTTTCCACTTCATCTTACCATTT
(mitochondrial DNA)	Reverse	TGTTGGGTTGTTTGATCCTG
β-actin	Forward	GGAAAAGAGCCTCAGGGCAT
(nuclear DNA)	Reverse	GAAGAGCTATGAGCTGCCTGA

Supplementary Table 4. Primer pairs used for semi-nested RT-PCR analysis

Transcript	PCR	Sequence
1 (AC097534.2)	1st	Forward 1: 5'-ACTCAGGAGAATTGGCGCTC-3'<br>Reverse A: 5'-GAAGTCGCTCGTCTCTCAGG-3'
	2nd	Forward 1<br>Reverse 1: 5'-CGCGTCTGTAATCCCAGCTA-3'
2 (TCONS_00009166)	1st	Forward 2: 5'-TGGAGAAGGGCGGAGTCATA-3'<br>Reverse A2: 5'-CTTGAATCTCCCATTAACAG-3'
	2nd	Forward 2<br>Reverse 2: 5'-GAGGCTGAGGCAGGAGAATC-3'
3 (TCONS_00008646)	1st	Forward 3: 5'-GCTGGTCGAATGTTCCCAGA-3'<br>Reverse A
	2nd	Forward 3<br>Reverse 3: 5'-ATATTAGCCACGCCCATCCC-3'
4 (TCONS_00008648)	1st	Forward 4: 5'-TGAGGGACGGAGGAACTACT-3'<br>Reverse A
	2nd	Forward 4<br>Reverse 4: 5'-CACGATCACTTCCCACACCA-3'
5 (TCONS_00008647)	1st	Forward 5: 5'-GGAGAGAGGGATGGGCGT-3'<br>Reverse A
	2nd	Forward 5<br>Reverse 5: 5'-GATTTTCTGCGTCGATGGCA-3'
6 (TCONS_00008649)	1st	Forward 6: 5'-GCTGGTCGAATGTTCCCAGA-3'<br>Reverse A
	2nd	Forward 6<br>Reverse 6: 5'-CGCGTCTGTAATCCCAGCTA-3'

Supplementary Table 5. Probe sets and primer pairs used for RAP analysis

5'-biotinylated antisense ssDNA probe set		Sequences
Hint1	1	5'-GCGGGAGCGCGCGGAGACTGCCGGCTCGCGCCCGGTGG GCCTGCGCGTGCGCACT-3'
	2	5'-CATCGCGGCCGCTCACTGTCCTTCCCGCGCCGCGGCCAC AGTGGGGAGAGGAACC-3'
	3	5'-CGAAGATCGTGTCGCCGCCGGGCTGGGCCACTTGAGCCT TGGCAATCTCGTCAGC-3'
	4	5'-CACCGGTCGTCCTCGAAGATGATCTTGGCGGGGATTTCTT TGCGGATGATCTTGC-3'
	5	5'-AATGTGATAGACAGACTGTCCCCCGTCTGCACCTTCATTCA CCACCATCCGGTAC-3'
	6	5'-ACCTGCTTTAACCAGGAGGCCAGTTCATCTGCCGACCCCC AAGGACATGGAGGTG-3'
LacZ	1	5'- GTGAGCGAGTAACAACCCGTCGGATTCTCCGTGGGAACA AACGGCGGATTGACCG-3'
	2	5'- GTAGTCACGCAACTCGCCGCACATCTGAACTTCAGCCTCC AGTACAGCGCGGCTG-3'
	3	5'- GGCTTCTGCTTCAATCAGCGTGCCGTCGGCGGTGTGCAG TTCAACCACCGCACGA-3'

Primer		Sequences
Caren	Forward	5'-GCATCATGACTGCCTGGG-3'
	Reverse	5'-ATTTGTGTGTTCCTGGTGGG-3'
Hint1	Forward	5'-CTGGTGATACCCAAGAAGCA-3'
	Reverse	5'-GATCTGCAGCACATTTCTTG-3'
