# Supplementary material for: The lncRNA Caren antagonizes heart failure by inactivating DNA damage response and activating mitochondrial biogenesis
Source: Nat Commun. 2021 May 5;12:2529. doi: 10.1038/s41467-021-22735-7 (PMC8099897; doi:10.1038/s41467-021-22735-7)
Supplement: Supplementary file 3 — Description of Additional Supplementary Files [file 41467_2021_22735_MOESM3_ESM.docx]

**Description of Additional Supplementary Files**

**File name: Supplementary Data 1.**

**Description: Echocardiography measurements at Fig. 2b, 3b, 7b, 8f, and Supplementary Fig. 5d, 12b, 13c–g.**

Mean and ± SD values for each parameter measured by echocardiography. The notation “;d” and “;s” indicate “at end of diastole” and “at end of systole”, respectively. Statistical significance was determined by one-way ANOVA with Sidak’s post hoc test (**Fig. 2b, 3b, 7b, and 8f**) or two-sided unpaired Student’s *t* -test (**Supplementary Fig. 5d, 12b, and 13c–g**). n.s; not significant, between groups.
