# Supplementary material for: The lncRNA Caren antagonizes heart failure by inactivating DNA damage response and activating mitochondrial biogenesis
Source: Nat Commun. 2021 May 5;12:2529. doi: 10.1038/s41467-021-22735-7 (PMC8099897; doi:10.1038/s41467-021-22735-7)
Supplement: Supplementary file 5 — Reporting Summary [file 41467_2021_22735_MOESM5_ESM.pdf]

nature research

Corresponding author(s): Yuichi Oike

Last updated by author(s): Feb 15, 2021

Reporting Summary

Nature Research wishes to improve the reproducibility of the work that we publish. This form provides structure for consistency and transparency in reporting. For further information on Nature Research policies, see our Editorial Policies and the Editorial Policy Checklist.

Statistics

For all statistical analyses, confirm that the following items are present in the figure legend, table legend, main text, or Methods section.

n/a	Confirmed	
☐	☒	The exact sample size (n) for each experimental group/condition, given as a discrete number and unit of measurement
☐	☒	A statement on whether measurements were taken from distinct samples or whether the same sample was measured repeatedly
☐	☒	The statistical test(s) used AND whether they are one- or two-sided<br>*Only common tests should be described solely by name; describe more complex techniques in the Methods section.*
☒	☐	A description of all covariates tested
☒	☐	A description of any assumptions or corrections, such as tests of normality and adjustment for multiple comparisons
☐	☒	A full description of the statistical parameters including central tendency (e.g. means) or other basic estimates (e.g. regression coefficient) AND variation (e.g. standard deviation) or associated estimates of uncertainty (e.g. confidence intervals)
☐	☒	For null hypothesis testing, the test statistic (e.g. F, t, r) with confidence intervals, effect sizes, degrees of freedom and P value noted<br>*Give P values as exact values whenever suitable.*
☒	☐	For Bayesian analysis, information on the choice of priors and Markov chain Monte Carlo settings
☒	☐	For hierarchical and complex designs, identification of the appropriate level for tests and full reporting of outcomes
☒	☐	Estimates of effect sizes (e.g. Cohen's d, Pearson's r), indicating how they were calculated

Our web collection on statistics for biologists contains articles on many of the points above.

Software and code

Policy information about availability of computer code

Data collection	Wave (2.4.1), FACSDiva (6.1), and FACSuite (1.0.6).
Data analysis	Vevo 2100 version 1.4, Multi Gauge software (3.1), Maxquant software (1.6.0.16), Perseus (1.6.0.7), Flowjo (version X), GraphPad Prism (version 7.03), MaxLFQ algorithm, STATA software (15.0), and Wave (2.4.1).

For manuscripts utilizing custom algorithms or software that are central to the research but not yet described in published literature, software must be made available to editors and reviewers. We strongly encourage code deposition in a community repository (e.g. GitHub). See the Nature Research guidelines for submitting code & software for further information.

Data

Policy information about availability of data

All manuscripts must include a data availability statement. This statement should provide the following information, where applicable:

- Accession codes, unique identifiers, or web links for publicly available datasets
- A list of figures that have associated raw data
- A description of any restrictions on data availability

Proteomic data presented in Fig. 4a and 5a have been deposited to the ProteomeXchange Consortium via the PRIDE partner repository (https://www.ebi.ac.uk/pride/) with an accession number PXD016186.
The following publicly available databases were used: STRING database (https://string-db.org) for performing enrichment analysis of differentially-expressed proteins, and ENCODE database (http://genome.ucsc.edu/ENCODE/index.html) for analyzing histone modifications of interest lincRNA genes and obtaining sequences of human lincRNA transcripts. All remaining data supporting the findings of this study are available within the article and its Supplementary Information files or from the corresponding author upon reasonable request. Source data are provided with this paper.

Field-specific reporting

Please select the one below that is the best fit for your research. If you are not sure, read the appropriate sections before making your selection.

☒ Life sciences ☐ Behavioural & social sciences ☐ Ecological, evolutionary & environmental sciences

For a reference copy of the document with all sections, see nature.com/documents/nr-reporting-summary-flat.pdf

Life sciences study design

All studies must disclose on these points even when the disclosure is negative.

Sample size	No statical methods were used to predetermine sample size for animal studies, but sample sizes were determined based on previous reports (Nat Commun 7: 13016, 2016 and Circ J 83: 368-378, 2019).
Data exclusions	No data were excluded from the analyses.
Replication	Replicates were used in in vivo experiments as noted in figure legends. In vitro experiments were performed at least three times independently. All attempts at replication were successful.
Randomization	In all the animal studies, mice were randomly assigned to the different groups and treated in a random order. In vitro experiments with cell lines were randomly assigned to the different groups for treatments and control. For some in vitro experiments, such as in vitro translation assay and RNA pull down assay, randomization is irrelevant.
Blinding	In all the animal studies, the investigators were blinded to group allocation during data collection and analysis. In vitro experiments were not blind because some of the results are quantitative, or not subjective. Blinding was not feasible in these experiments.

Reporting for specific materials, systems and methods

We require information from authors about some types of materials, experimental systems and methods used in many studies. Here, indicate whether each material, system or method listed is relevant to your study. If you are not sure if a list item applies to your research, read the appropriate section before selecting a response.

Materials & experimental systems

n/a	Involved in the study	
☐	☒	Antibodies
☐	☒	Eukaryotic cell lines
☒	☐	Palaeontology and archaeology
☐	☒	Animals and other organisms
☐	☒	Human research participants
☒	☐	Clinical data
☒	☐	Dual use research of concern

Methods

n/a	Involved in the study	
☒	☐	ChIP-seq
☐	☒	Flow cytometry
☒	☐	MRI-based neuroimaging

Antibodies

Antibodies used	anti-phosphorylated ATM (Ser1981) (10H11.E12, #4526, Cell Signaling Technology), anti-ATM (D2E2, #2873, Cell Signaling Technology), anti-HINT1 (ab124912, Abcam), anti-HMGB2 (ab67282, Abcam), anti-SAP30 (ab125187, Abcam), anti-HA-tag (C29F4, #3724, Cell Signaling Technology), anti-TFAM (ab131607, Abcam), anti-Total OXPHOS Rodent Antibody Cocktail (ab110413, Abcam) containing the antibodies against NDUFB8 (Complex I, ab110242), SDHB (Complex II, ab14714), UQCRC2 (Complex III, ab14745), MTOCI (Complex IV, ab14705), and ATP5A (Complex V, ab14748), anti-NDUFB8 (Complex I, ab110242), anti-SDHB (Complex II, ab14714), anti-UQCRCI (Complex III, ab110252), anti-MTOCI (Complex IV, ab14705), and anti-ATP5A (Complex V, ab14748), anti-Hsc70 (sc-7298, Santa Cruz Biotechnology), anti-UQCRCI (Complex III, ab110252, Abcam), HRP-conjugated donkey anti-rabbit IgG (NA934A, GE Healthcare Life Science), or HRP-conjugated sheep anti-mouse IgG (NA9310V, GE Healthcare Life Science) .
Validation	Validation statements of manufacturers' website (Abcam, Cell Signaling Technology, or Santa Cruz Biotechnology).<br>anti-phosphorylated ATM (Ser1981): https://www.cellsignal.com/products/primary-antibodies/phospho-atm-ser1981-10h11-e12-mouse-mab/4526<br>anti-ATM: https://www.cellsignal.com/products/primary-antibodies/atm-d2e2-rabbit-mab/2873<br>anti-HINT1: https://www.abcam.com/hint1-antibody-epr5108-ab124912.html<br>anti-HMGB2: https://www.abcam.com/hmgb2-antibody-ab67282.html<br>anti-SAP30: https://www.abcam.com/sap30-antibody-ab125187.html<br>anti-HA-tag: https://www.cellsignal.com/products/primary-antibodies/ha-tag-c29f4-rabbit-mab/3724<br>anti-TFAM: https://www.abcam.com/mttfa-antibody-mitochondrial-marker-ab131607.html<br>anti-Total OXPHOS Rodent Antibody Cocktail: https://www.abcam.com/total-oxphos-rodent-wb-antibody-cocktail-ab110413.html

anti-NDUFB8 (Complex I, ab110242): https://www.abcam.com/ndufb8-antibody-20e9dh10c12-ab110242.html
anti-SDHB: https://www.abcam.com/sdhb-antibody-21a11ae7-ab14714.html
anti-UQCRCI: https://www.abcam.com/ubiquinol-cytochrome-c-reductase-core-protein-i-antibody-16d10ad9ah5-ab110252.html
anti-MTOCI: https://www.abcam.com/mtco1-antibody-1d6e1a8-ab14705.html
anti-ATP5A: https://www.abcam.com/atp5a-antibody-15h4c4-mitochondrial-marker-ab14748.html
anti-Hsc70: https://www.scbt.com/p/hsc-70-antibody-b-6?productCanUrl=hsc-70-antibody-b-6&_requestid=3009559

Eukaryotic cell lines

Policy information about cell lines

Cell line source(s)	LentiX-293 cells and human iPS cell-derived cardiomyocytes were purchased from Takara Bio Inc. Rat cardiomyocyte cell line H9c2 and mouse myoblast cell line C2C12 were obtained from ATCC. Mouse embryonal carcinoma cell line P19.CL6 was obtained from RIKEN BRC.
Authentication	None of the cell lines used were authenticated.
Mycoplasma contamination	Cell lines were not tested for mycoplasma contamination.
Commonly misidentified lines (See ICLAC register)	No commonly misidentified cell lines were used in this study.

Animals and other organisms

Policy information about studies involving animals; ARRIVE guidelines recommended for reporting animal research

Laboratory animals	All mice were in the C57BL/6N background. Male 10–12 weeks old alphaMHC-EGFP Tg, Caren beta-geo/beta-geo, CAG-Caren Tg, CAG-Caren-(fragment A-E) Tg, alphaMHC-Caren Tg, and Hint1 tm1a(EUCOMM)Wtsi (Hint1-/-) mice were used. All animals were fed a normal diet (ND; CE-2, CLEA, Tokyo, Japan), bred in a mouse house with automatically controlled lighting (12 h on, 12 h off), maintained at a stable temperature of 22 ± 2ºC and a relative humidity of 40-80%.
Wild animals	No wild animals were used in this study.
Field-collected samples	No field-collected samples were used in this study.
Ethics oversight	All experimental procedures were approved by the Kumamoto University Ethics Review Committee for Animal Experimentation (approval No. A27-063, A29-072 and A2019-063).

Note that full information on the approval of the study protocol must also be provided in the manuscript.

Human research participants

Policy information about studies involving human research participants

Population characteristics	Heart tissues obtained from 9 autopsy cases from humans (4 men, 5 women; mean age ± s.e.m., 67.8 ± 19.9 years) who died of various cases shown in Supplementary Table 1.
Recruitment	No patients were recruited specifically for this study. No self-selection is involved.
Ethics oversight	The study protocol was approved by the Ethics Committees of Kumamoto University (approval No. 1454).

Note that full information on the approval of the study protocol must also be provided in the manuscript.

Flow Cytometry

Plots

Confirm that:

- [x] The axis labels state the marker and fluorochrome used (e.g. CD4-FITC).
- [x] The axis scales are clearly visible. Include numbers along axes only for bottom left plot of group (a 'group' is an analysis of identical markers).
- [x] All plots are contour plots with outliers or pseudocolor plots.
- [x] A numerical value for number of cells or percentage (with statistics) is provided.

Methodology

Sample preparation	To prepare cardiomyocytes and non-cardiomyocytes, ventricles were harvested from 10-week-old male Tg mice overexpressing enhanced green fluorescent protein (EGFP) driven by the murine alphaMHC (Myh6) promoter (aplhaMHC-EGFP), and tissue was minced and digested with 0.075% collagenase, 0.12% trypsin and 0.02% DNase at 37°C for 40 min. Cells were collected, resuspended and then passed through a 100-um mesh filter into 50-ml centrifuge tubes.

	To determine mitochondrial membrane potential and mitochondrial ROS levels, cultured cells were treated with 200 nM TMRM (Thermo Fisher Scientific Inc) and 5 uM MitoSOX Red Superoxide Indicator (Thermo Fisher Scientific Inc), respectively. Cells were treated with dispase and harvested.
Instrument	Cell sorter FACSAria II and BD FACSVerse.
Software	FACSDiva and FACSuite were used to collect the flow cytometry data. Flowjo was used to analyze the flow cytometry data.
Cell population abundance	The purity of sorted GFP+ cells (cardiomyocytes) and GFP- cells (non-cardiomyocytes) is more than 99%.
Gating strategy	1. Gate out debris on FSC and SSC plot. 2. Gate on single cells (FSC-A/FSC-H). 3. Gate out dead cells. 4. Gate on population of interest as using a negative sample (e.g. cardiomyocytes from heart of WT mice or cultured cells untreated with TMRM and MitoSOX Red).

☒ Tick this box to confirm that a figure exemplifying the gating strategy is provided in the Supplementary Information.
